# Alterations of resting-state networks of Parkinson‘s disease patients after subthalamic DBS surgery

**DOI:** 10.1016/j.nicl.2023.103317

**Published:** 2023-01-04

**Authors:** Matthias Sure, Sean Mertiens, Jan Vesper, Alfons Schnitzler, Esther Florin

**Affiliations:** a Institute of Clinical Neuroscience and Medical Psychology, Medical Faculty, Heinrich-Heine University, Düsseldorf, Germany; b Department of Functional Neurosurgery and Stereotaxy, Medical Faculty, University Hospital, Düsseldorf, Germany; c Department of Neurology, Center for Movement Disorders and Neuromodulation, Medical Faculty, University Hospital, Düsseldorf, Germany

**Keywords:** BGC, Basal ganglia-cortical, DBS, Deep brain stimulation, FON, Fronto-occipital network, HC, Healthy controls, HPI, Head position indicator, UPDRS III, Unified Parkinson's Disease Rating Scale motor score, MEG, Magnetoencephalography, PAC, Phase-amplitude coupling, PD, Parkinson’s disease, SSP, Singal-space projector, STN, Subthalamic nucleus, RSN, Resting-state network, Resting-state network, PAC, MEG, PD, Stun effect

## Abstract

The implantation of deep brain stimulation (DBS) electrodes in Parkinson’s disease (PD) patients can lead to a temporary improvement in motor symptoms, known as the stun effect. However, the network alterations induced by the stun effect are not well characterized. As therapeutic DBS is known to alter resting-state networks (RSN) and subsequent motor symptoms in patients with PD, we aimed to investigate whether the DBS-related stun effect also modulated RSNs.

Therefore, we analyzed RSNs of 27 PD patients (8 females, 59.0 +- 8.7 years) using magnetoencephalography and compared them to RSNs of 24 age-matched healthy controls (8 females, 62.8 +- 5.1 years). We recorded 30 min of resting-state activity two days before and one day after implantation of the electrodes with and without dopaminergic medication. RSNs were determined by use of phase-amplitude coupling between a low frequency phase and a high gamma amplitude and examined for differences between conditions (i.e., pre vs post surgery).

We identified four RSNs across all conditions: sensory-motor, visual, fronto-occipital, and frontal. Each RSN was altered due to electrode implantation. Importantly, these changes were not restricted to spatially close areas to the electrode trajectory.

Interestingly, pre-operative RSNs corresponded better with healthy control RSNs regarding the spatial overlap, although the stun effect is associated with motor improvement.

Our findings reveal that the stun effect induced by implantation of electrodes exerts brain wide changes in different functional RSNs.

## Highlights

• Resting state networks in Parkinson patients are altered by the stun effect.
• Higher overlap with healthy resting state networks before surgery.
• No link between alterations in resting state networks and Parkinson symptom severity.

## 1 Introduction

Parkinson’s disease (PD) is a neurodegenerative disorder that is characterized mainly by motor symptoms (Poewe et al., 2017). The neuropathological hallmark of PD is the degeneration of dopaminergic neurons in the substantia nigra pars compacta (Mullin and Schapira, 2015). As PD progresses motor fluctuations occur and symptoms cannot be well controlled by medical treatment. In these cases, deep brain stimulation (DBS) of the subthalamic nucleus (STN) is an option to alleviate motor symptoms (Deuschl et al., 2006). It is known, that the sole implantation of the electrodes into STN without stimulation already leads to a temporary alleviation of symptoms. This effect is called stun effect and is probably caused by inflammatory processes and edema (Chen et al., 2006, Mann et al., 2009). To date, only one prior fMRI study has revealed altered cortical and subcortical functional connectivity due to the stun effect (Luo et al., 2021). Thus, detailed investigation of changes in functional networks is missing.

Analyzing electrophysiological resting-state networks (RSN) should lead to a better understanding of the stun effect. Changes in functional networks represent a prime candidate measure, as altered RSNs have been associated with PD (de Schipper et al., 2018, Göttlich et al., 2013) and with dopaminergic medication RSNs of PD patients align better with the RSNs of healthy controls (Schneider et al., 2020). In addition, DBS modulates spatially and spectrally segregated STN – cortical RSNs in PD patients (Oswal et al., 2016). Furthermore, RSN alterations have been related to PD symptoms, cognitive impairment (Tessitore et al., 2012b), and freezing of gait (Tessitore et al., 2012a).

It has been shown that brain activity at rest is organized in different hierarchically structured functional networks (Cordes et al., 2002, Doucet et al., 2011) that are associated with a range of motor and cognitive tasks (Smith et al., 2009, Thomas Yeo et al., 2011). Thus, analysis of RSNs provides the opportunity to study brain network alterations without subjecting patients to complex motor or cognitive tasks.

The stun effect is of clinical relevance because its occurrence has been linked to the subsequent effectiveness of DBS (Maltête et al., 2008, Tykocki et al., 2013), although reports on this are inconsistent (Granziera et al., 2008). To better understand the link between stun effect and DBS effect, it is eminent to investigate which functional areas are affected by the stun effect. Therefore, the question arises whether the stun effect is related to the areas impaired by the electrode trajectory.

If the stun effect modulates functional networks, the electrode position-specific side effects of DBS could be described even better. In a further step, stimulation parameter-dependent side effects (Dayal et al., 2017, Hartmann et al., 2019) could be optimally linked to RSN changes induced by DBS electrode implantation. Thus, initial DBS settings could be optimized based on RSN changes induced by the stun effect.

To tackle these questions, we recorded the cortical activity of 27 PD patients via magnetoencephalography (MEG) both before (in the following: pre) and after (in the following: post) DBS surgery. On each measurement day, patients were recorded at rest OFF-stimulation once OFF- and once ON-medication for 30 min. We identified four networks present in each of the four conditions: sensory-motor, visual, frontal, and fronto-occipital. RSN connectivity measures for each network were compared between the pre- and post-measurement for both medication states.

The megPAC approach used here is one of many methods (e.g., amplitude correlation, spectral coherence, and autoregressive models; Colclough et al., 2016, Florin and Baillet, 2015) that can determine networks based on cortical time series determined via MEG. The phase-amplitude coupling (PAC; Özkurt and Schnitzler, 2011) used here is particularly suitable for determining RSNs in the context of PD patients, as the PAC correlates positively with PD motor symptoms and can also be reduced by therapeutic interventions (De Hemptinne et al., 2015, De Hemptinne et al., 2013, Tanaka et al., 2022). Furthermore, RSNs based on PAC have been shown to be comparable to fMRI-RSNs (Pelzer et al., 2021).

As the stun effect has been related to the alleviation of motor symptoms in PD, we hypothesized that the sensory-motor RSN will be altered due to electrode implantation, and further, that these implantation-related changes in the sensory-motor RSN will be linked to changes in clinical scores of motor symptom severity. Due to the wide-spread effects of DBS, we also expect the stun effect to induce alterations within RSNs not traditionally linked to the electrode trajectory (i.e., outside sensory-motor brain areas).

## 2 Methods

### 2.1 Subjects and surgery

A total of 27 (8 female) PD patients (age: 59.0 +- 8.7 years [mean +- std]) were included in this study (see Table 1). The patients were identical to the sample reported in Sure et al. (2021). Furthermore, a control group of 24 (8 female) healthy age-matched participants (HC; 62.8 +- 5.1 years) was recruited. Written informed consent was obtained from all patients and HC subjects prior to study participation. Patients had been selected for DBS treatment according to the guidelines of the German Society for Neurology (Deutsche Gesellschaft für Neurologie e.V., 2016). The study was approved by the local ethics committee (study no. 5608R) and conducted in accordance with the Declaration of Helsinki.

**Table 1:** Clinical details.

Subject	Age	Sex	UPDRS pre-OFF	UPDRS pre-ON	UPDRS post-OFF	UPDRS post-ON	Days before/after surgery	Electrode	Levodopa dose [mg] pre/post
1	68	male	–	41	48	38	2/1	Abbott Infinity	125/250
2	56	male	62	28	25	23	22/1	Abbott Infinity	150/150
3	64	male	43	37	31	25	2/1	Abbott Infinity	200/200
4	62	female	27	12	22	24	1/4	Abbott Infinity	200/200
5	69	male	44	37	33	32	3/4	Abbott Infinity	150/150
6	45	male	22	13	22	10	2/1	Abbott Infinity	200/200
7	55	male	33	21	21	18	2/1	Abbott Infinity	200/200
8	77	male	46	17	58	–	2/2	Abbott Infinity	200/200
9	54	male	40	14	–	9	2/1	Abbott Infinity	150/150
10	60	female	22	21	32	28	2/1	Abbott Infinity	200/150
11	46	male	21	9	15	11	141/1	Abbott Infinity	200/150
12	56	male	45	43	31	19	2/1	Boston Scientific	150/150
13	58	male	54	26	25	14	2/1	Abbott Infinity	150/150
14	67	male	28	16	21	12	2/1	Abbott Infinity	200/200
15	54	male	49	45	37	27	2/1	Abbott Infinity	150/200
16	41	male	35	7	34	16	2/1	Abbott Infinity	300/300
17	58	female	23	8	25	13	2/2	Abbott Infinity	100/50
18	65	female	32	17	20	12	2/1	Abbott Infinity	100/100
19	72	female	28	19	42	35	2/1	Abbott Infinity	150/150
20	44	female	59	16	42	14	3/3	Abbott Infinity	225/225
21	68	male	38	20	49	27	2/3	Abbott Infinity	Best med on/
22	53	male	30	14	17	8	3/1	Abbott Infinity	150/150
23	58	male	41	24	33	25	1/1	Abbott Infinity	150/
24	69	male	42	31	33	29	10/1	Abbott Infinity	250/200
25	62	female	33	18	26	17	2/1	Abbott Infinity	150/150
26	55	female	39	32	43	30	2/1	Abbott Infinity	150/150
27	58	male	36	17	14	11	2/1	Abbott Infinity	Best med on/150

The measurements prior to DBS electrode implantation were usually acquired two days before surgery (2, 1–141 days [median, range]. Of note, surgery had to be rescheduled for 2 of the 27 patients, which may lead to confounding variability due to discrepant surgical timelines. To address this concern, these two patients were excluded from all subsequent analyses (time prior to DBS surgery for remaining cohort: 2, 1–3 [median, range]). Measurements after DBS electrode implantation were acquired one day after surgery (1, 1–4 days, [median, range]). The DBS electrodes were bilaterally implanted in the dorsal part of the subthalamic nucleus (STN) at the Department of Functional Neurosurgery and Stereotaxy in Düsseldorf. In one case, the Boston Scientific Vercise segmented lead (Boston Scientific Corporation, Marlborough, MA, USA) was implanted, and in all other cases the St. Jude Medical Directional lead 6172 (Abbott Laboratories, Lake Bluff, IL, USA). During the measurements, electrodes were externalized using the St. Jude Medical Directional extension 6373 (Abbott Laboratories, Lake Bluff, IL, USA) and connected to the EEG amplifier integrated in a 306 channel MEG system (Elekta Neuromag, Helsinki, Finland). No electrical stimulation was applied during any of the measurements.

### 2.2 Experimental setup and recordings

The MEG measurement took place in a magnetically shielded room. Patients were seated in the MEG and remained at rest with their eyes open. During the entire measurement, the patients were asked to look at a black fixation cross on a white poster. An eye tracker (iView X 2.2, SensoMotoric Instruments, Teltow, Germany) was used to ensure that the eyes were consistently kept open. On each of the two measurement days, there were three consecutive measurement blocks of 10 min OFF medication and three 10-minute blocks ON medication, resulting in a total measurement duration of 120 min per patient (60 min for each pre and post electrode implantation). To ensure that patients were in their medication OFF state, oral PD medication was discontinued overnight for at least 12 h. For patients with an apomorphine pump, the pump was stopped at least 1 h before the start of the measurement. After the OFF measurements, patients received a standardized dose of fast-acting soluble levodopa (1.5 times their morning levodopa dose). To ensure a stable medication ON state, we waited until a significant clinical improvement occurred, i.e., for at least 30 min after ingestion. The Unified Parkinson's Disease Rating Scale (UPDRS III) motor score was assessed for each of the 30-minute measurement blocks and tested for differences between the recording conditions (i.e., pre-OFF, pre-ON, post-OFF, and post-ON) using a paired sample *t*-test.

The MEG data were recorded with a sampling frequency of 2400 Hz and a low-pass filter of 800 Hz. To identify artifacts caused by heartbeat or eye movements, an electrocardiogram and an electrooculogram were acquired. To determine the relative position of the MEG sensors to the brain, four head position indicator (HPI) coils were placed on the subject’s head and digitized via the Polhemus system (Polhemus Isotrack, Colchester, VT, USA). For subsequent co-registration with individual T1 weighted anatomical MRI scans (depending on scanner availability: 3T Trio Tim, 3T Prisma, 1.5T Avanto, 1.5T Avanto-fit, 1.5T Sola; all with 1 mm^3^ voxel size), 100 points distributed over the skull surface were additionally digitized.

### 2.3 Signal processing

Preprocessing and further analysis of the MEG data was done using Matlab (version R 2016b; The MathWorks, Inc., Natick, MA, USA) and the Matlab based toolbox Brainstorm (http://neuroimage.usc.edu/brainstorm/Introduction; Tadel et al., 2011).

Identification of artifacts was independently performed by two people. If no consensus was achieved for a time segment, this time segment was considered artefactual and discarded from further analysis. The signal-space projectors (SSP) supplied by the Neuromag system were applied by default.

In addition, frequent artifacts following the same pattern like eye blinks or cardiac artifacts were removed via custom-made SSPs. Time periods with irregular artifacts like movement artifacts were removed from all channels. Due to excessive artifacts, the data of two patients had to be excluded for the pre-OFF and pre-ON conditions. In addition, the data of one further patient had to be excluded for all four conditions.

Subsequently, time series activity of the artifact-cleaned sensor data was projected to the source level. For this purpose, the cortical surfaces of the individual T1-MRI images were extracted for each patient using FreeSurfer (http://freesurfer.net, v.5.3.0). The individual cortex sheet was down-sampled to ∼15000 vertices within brainstorm. The MEG sensor data and the anatomical data of each patient were transformed into one coordinate system in Brainstorm using the Polhemus data as well as the anatomical landmarks (nasion, left and right pre-auricular point). The forward problem was solved via the overlapping spheres method implemented in Brainstorm (Huang et al., 1999). The lead-fields were computed based on one elementary current dipole for each vertex with an orientation perpendicular to the cortex. For the inverse solution, the linearly constrained minimum variance beamformer implemented in Brainstorm was employed (Van Veen et al., 1997). The necessary individual data covariance matrix was calculated for each measurement block and a separate noise covariance matrix for each measurement day. The separate noise covariance matrix was determined from a 5-minute empty room recording at the end of each measurement day after subject recordings.

### 2.4 Resting-state network estimation

To determine electrophysiological RSNs, we chose the data-driven megPAC approach of Florin and Baillet (2015) which is based on the concept of a synchronized gating between low- and high-frequency information and has been shown to be capable of matching fMRI-RSNs (Pelzer et al., 2021). First, PAC is calculated for each reconstructed cortical source. Then the frequency pair with the highest coupling between the low-frequency phase from 2 to 30 Hz and the high-frequency amplitude from 80 to 150 Hz, is determined. Next, the amplitude of the high-frequency signal is interpolated between the peaks and troughs of the low-frequency signal, resulting in a new time series referred to as megPAC. This time series is generated for each cortical source in the individual anatomy-based source space, sampled down to 10 Hz, and projected onto the standard ICBM152 brain with 15,002 vertices using FreeSurfer's spherical registration (Fonov et al., 2011). On the standard brain, the megPAC time series is spatially smoothed using a Gaussian kernel with a full width at half maximum of 7 mm as implemented in Brainstorm. Downsampling and spatial smoothing were performed to increase comparability with the data processing of more commonly used fMRI-RSNs (e.q., Brookes et al., 2011) and to align with the original methods of Florin and Baillet (2015). To extract the group-level RSNs, the megPAC time series of all available patients are concatenated and the correlation between all cortical time series are calculated, resulting in a 15,002 by 15,002 correlation matrix. This dimensionality of this matrix was reduced by using a subset of 1175 evenly distributed sources over the cortical surface template. Based on noise data with the same length as the original data and the same preprocessing steps, a projector was defined to remove noise from the correlation matrix. Finally, using a singular value decomposition, the RSNs were determined as principal modes of this noise cleaned correlation matrix. This resulted in cortical maps with values between 0 and 1, which are further referred to as coupling strength. For further details on the computation please refer to Florin and Baillet (2015). We then selected the 10 principal components that explained the most variability in the data.

### 2.5 Assignment of RSNs of interest

**Fig. 1:**
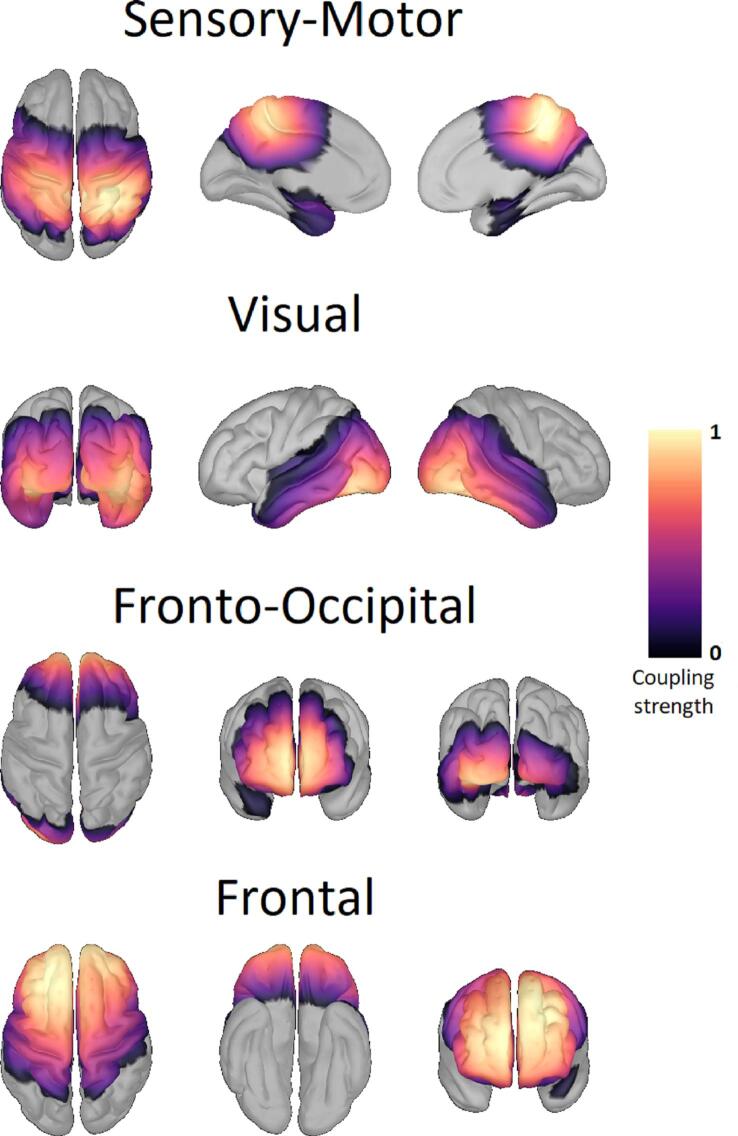
Template resting-state networks based on healthy controls Shown here are the four template resting-state networks (RSN) of the healthy controls. The same RSNs are also found in the four PD conditions. The rows display the sensory-motor, visual, fronto-occipital, and frontal RSN. The color scale marks the coupling strength from 0 to 1, with a warmer color indicating a higher coupling strength. No threshold was applied.

We used the megPAC approach to calculate RSNs for each condition: pre-OFF, pre-ON, post-OFF, and post-ON. Within the set of ten RSNs computed for each condition, we identified four RSNs of interest consistently in all conditions: the sensory-motor, visual, frontal, and fronto-occipital network (FON). The key criterion for selecting these four RSNs was that they were present in all conditions. To support this selection of RSNs, we also successfully identified these four RSNs in an age-matched control group (Fig. 1), which highlights the physiological significance of these RSNs.

To compare the RSNs between the recording conditions we used a Jackknife approach with a leave one out technique (Stute, 1996). The RSNs were calculated for each condition separately. For a condition with N patients, we performed N Jackknife runs, omitted one patient at a time. This results in N sets containing ten RSNs each. To determine changes for a given RSN between conditions, the corresponding RSN had to be assigned in the Jackknife runs. The assignment of the four RSNs of interest from each Jackknife run was done separately for each recording condition. As templates for this assignment, we used the RSNs of each condition determined across all participants. For the four template RSNs of each condition, the best matching network was determined for each Jackknife run of the corresponding recording condition. For the purpose of assignment, the cortical map of both the template RSNs and the Jackknife RSNs were thresholded at 40 % of the maximum coupling strength for comparability with the methods of Florin and Baillet (2015). This results in a binary vertex mask.

To quantify the correlation between the binary vertex masks of each of the four template RSNs and the ten RSNs of a Jackknife run, we employ the Phi coefficient also known as Mean Square Contingency Coefficient (Yule, 1912). Based on the Phi coefficient, a one-to-one matching was made between the RSNs of a jackknife run and the four template RSNs. Assignment was made with a minimum phi coefficient of 0.6 (RSN: mean phi coefficient +- std, minimum; Sensory-Motor: 0.95 +- 0.03, 0.79; Visual: 0.96 +- 0.03, 0.80; Frontal: 0.94 +- 0.07, 0.62; FON: 0.96 +- 0.03, 0.88).

### 2.6 Statistical testing between RSNs of the pre- and post-condition

Based on this assignment, the RSNs of the Jackknife runs of the pre- and post-condition of the same RSN were statistically compared with a two-tailed unequal variance *t*-test, separately for both medication states.

To compare across conditions, we performed the following steps:

1. Separately for both conditions a pseudo value $\Delta_{i}$ was calculated for a RSN of the i-th Jackknife run $\Theta_{i}$

$$\Delta_{i}=N\cdot \Theta_{0}-\left(N-1\right)\cdot \Theta_{i}$$

Where $\Theta_{0}$ is the baseline estimator without leaving one out.

2. The set of N pseudo values allows computing the mean value

$$\overset{\wedge }{\Delta }=\frac{1}{N}\cdot \sum_{i=1}^{n}\Delta_{i}$$

and its the standard deviation

$$\sigma =\frac{1}{N-1}\cdot \sum_{i=1}^{n}{\left(\Delta_{i}-\overset{\wedge }{\Delta }\right)}^{2}$$

3. Step 2 allows evaluating significance of the difference across conditions 1 and 2 via a *t*-test:

$$t=\frac{{\overset{\wedge }{\Delta }}_{1}-{\overset{\wedge }{\Delta }}_{2}}{\sqrt{\frac{{\overset{\wedge }{\sigma }}_{1}}{N_{1}}-\frac{{\overset{\wedge }{\sigma }}_{2}}{N_{2}}}}$$

4. The associated p-value follows from Student’s t cumulative distribution function:

$$p=2\cdot \left(1-tcdf\left(t,dof\right)\right)$$

with degrees of freedom dof:

$$\mathrm{dof}=\frac{\left(N_{1}-1\right)\cdot \left(N_{2}-1\right)}{\left(N_{2}-1\right)\cdot C^{2}+{\left(1-C\right)}^{2}\cdot \left(N_{1}-1\right)}$$

where

$$C=\frac{\frac{\sigma_{1}^{2}}{N_{1}}}{\frac{\sigma_{1}^{2}}{N_{1}}+\frac{\sigma_{2}^{2}}{N_{2}}}$$

This comparison was calculated separately for each vertex within each RSN, i.e. only vertices belonging to a specific RSN were considered. Following (Florin and Baillet, 2015), we only considered vertices with a coupling strength of at least 0.4 in at least one of the two RSNs to be compared. The p-values determined for these vertices were corrected for the number of vertices, the four RSNs, and the four conditions using the Bonferroni correction implemented in Brainstorm. Reported results are significant after correction at the 0.05 level.

### 2.7 Overlap of PD RSNs with healthy RSNs

To quantitatively assess whether electrode implantation brings the RSNs closer to the physiological state, we evaluated the spatial correspondence between the PD RSNs of the individual jackknife runs in the four conditions and the corresponding HC RSNs based on threshold-constrained coupling strength. The spatial overlap was estimated using the Phi coefficient, which was calculated for each Jackknife run in each condition and the corresponding HC RSNs. For this the coupling strength of the RSNs were again thresholded at 40 %. Using a two-tailed *t*-test and Bonferroni correction, we examined whether there were significant condition-wise differences in the Phi coefficients between pre- and post-surgery PD RSNs and HC RSNs.

### 2.8 Comparison of the low and high PAC frequencies

Next, we tested whether the driving frequencies in PAC differed between conditions, as alterations of the dopaminergic tone can lead to frequency shifts in beta oscillations (Iskhakova et al., 2021). For this purpose, we determined the low and the high frequency based on the highest PAC value from each vertex of each patient and PD condition.

We then examined the low and high frequencies within the four RSNs. Here, separately for each condition, we selected all vertices that belonged to each of the RSN of interest which were determined across all subjects within the same condition. For each RSN of interest, the median of the low and high frequency was determined for each patient in each condition. Subsequently, a 3-factor ANOVA was calculated separately for the low and the high frequencies with the following factors: RSN, medication state, and electrode implantation. For this purpose, Matlab's implementation with associated post-hoc analysis including Bonferroni correction was used.

Finally, we also investigated the total spectral power between the pre-and post-recordings in five standard frequency bands (delta: 1–4 Hz, theta: 4–8 Hz, alpha: 8–12 Hz, beta: 12–35 Hz, gamma: 35–100 Hz). For this purpose, power spectra were calculated at the source level for each reconstructed time series using Welch’s method (Welch, 1967). A window length of 4 s with an overlap of 50 % was selected. These power spectra were projected onto a standard brain (ICBM 152). To ensure better comparability between runs, the 1/f characteristics of the power spectra were corrected by multiplying each power value with the corresponding frequency and normalized to the total power of 1–45 Hz and 55–95 Hz. To limit dimensionality, the first principal component of all vertices belonging to a scout from the Mindboggle atlas (Klein et al., 2017) was only considered in the further analysis. Power differences between the conditions were determined with an independent *t*-test with subsequent Bonferroni correction for 62 scouts and 5 frequency bands.

Furthermore, the local maximum was obtained for each frequency band in the power spectra. We tested for a shift in peak frequency due to electrode implantation with an independent *t*-test with subsequent Bonferroni correction.

## 3 Results

### 3.1 Patient characteristics

We assessed the UPDRS-III score in each recording condition. After Bonferroni correction, the UPDRS-III score was significantly higher in the pre-OFF condition (37.45 +- 11.11) compared to the pre-ON condition (22.30 +- 10.71, p = 1.72e-7, t = 7.74, df = 25). Similarly, scores were higher in the post-OFF condition (30.96 +- 11.49) compared to the post-ON condition (20.61 +- 9.07, p = 3.45e-6, t = 6.57, df = 24). Notably, there was a significant reduction in UPDRS-III score from the pre-OFF condition to the post-OFF condition (p = 0.04, t = -2.81, df = 24), which can be attributed to the stun effect. In contrast, there was no significant difference between pre-ON and post-ON.

### 3.2 Changes in RSNs

**Fig. 2:**
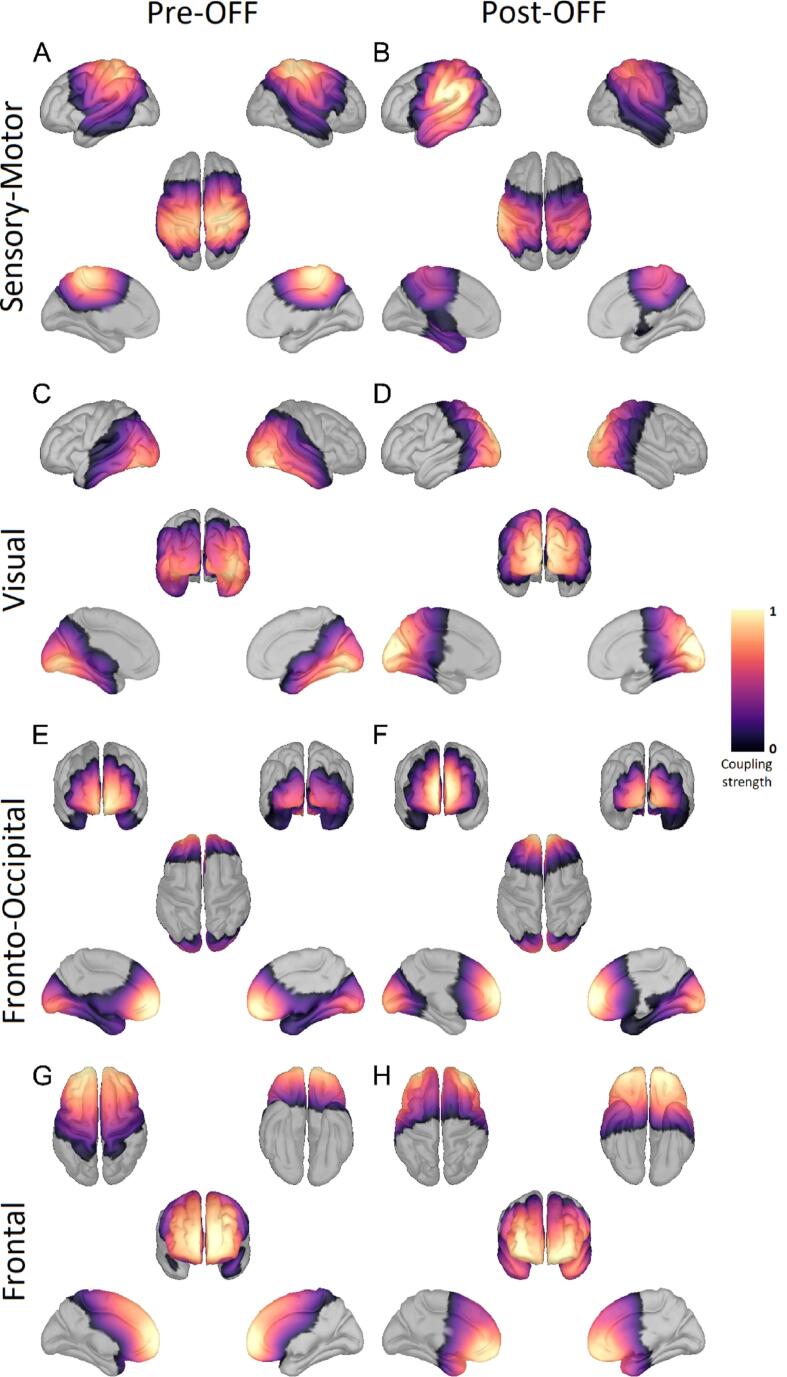
Resting-state networks based on Parkinson patients OFF medication Shown here are the four resting-state networks (RSN) determined across all PD patients OFF medication before electrode implantation (left column) and after electrode implantation (right column). The rows display the sensory-motor, visual, fronto-occipital, and frontal RSN. The color scale marks the coupling strength from 0 to 1, with a warmer color indicating a higher coupling strength. These RSNs were also found ON medication and in the HC data. No threshold was applied.

**Fig. 3:**
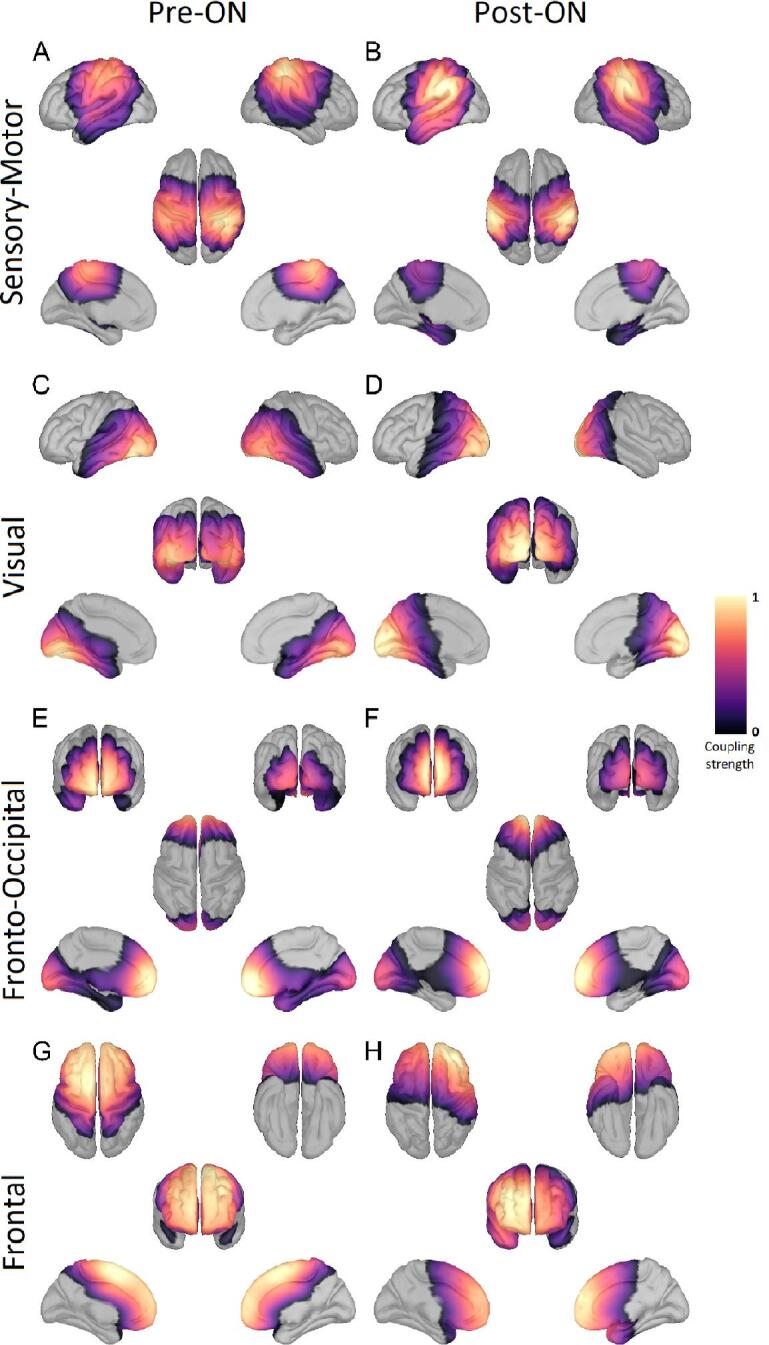
Resting-state networks based on Parkinson patients ON medication Shown here are the four resting-state networks (RSN) determined across all PD patients ON medication before electrode implantation (left column) and after electrode implantation (right column). The rows display the sensory-motor, visual, fronto-occipital, and frontal RSN. The color scale marks the coupling strength from 0 to 1, with a warmer color indicating a higher coupling strength. These RSNs were also found OFF medication and in the HC data. No threshold was applied.

Across all patients and healthy controls, we identified four distinct RSNs of cortical activity (Fig. 1, Fig. 2, Fig. 3). The sensory-motor RSN extended from the precentral gyrus over the postcentral and paracentral gyrus to the precuneus, superior parietal lobules, and supramarginal gyrus. Also, parts of the temporal lobe and cingulate gyrus were included in the sensory-motor RSN. The visual network was centered on the lateral occipital sulcus and traversed across the cuneus, precuneus, inferior and superior parietal lobule, the parahippocampal, lingual, and fusiform gyrus, as well as the pericalcarine cortex, and parts of the temporal lobe. The frontal network extended from the orbitofrontal gyrus across the inferior, medial, and superior frontal gyri to the paracentral, precentral, and postcentral gyri. Furthermore, it also includes parts of the cingulate gyrus. The fronto-occipital network (FON) spanned across the orbitofrontal cortex, rostral parts of the inferior, medial, and superior frontal gyrus, as well as the cingulate gyrus. The occipital area of the FON included lateral occipital sulcus, the lingual, fusiform, and pericalcarine gyrus. For each of these RSNs, we investigated the influence of the stun effect on the coupling strength of the RSNs and furthermore tested if the spatial overlap of the RSNs with HC RSNs differed between the PD conditions. We did not find any correlation between the changes in PD RSNs and changes in UPDRS-III scores.

### 3.3 Sensory-motor network

**Fig. 4:**
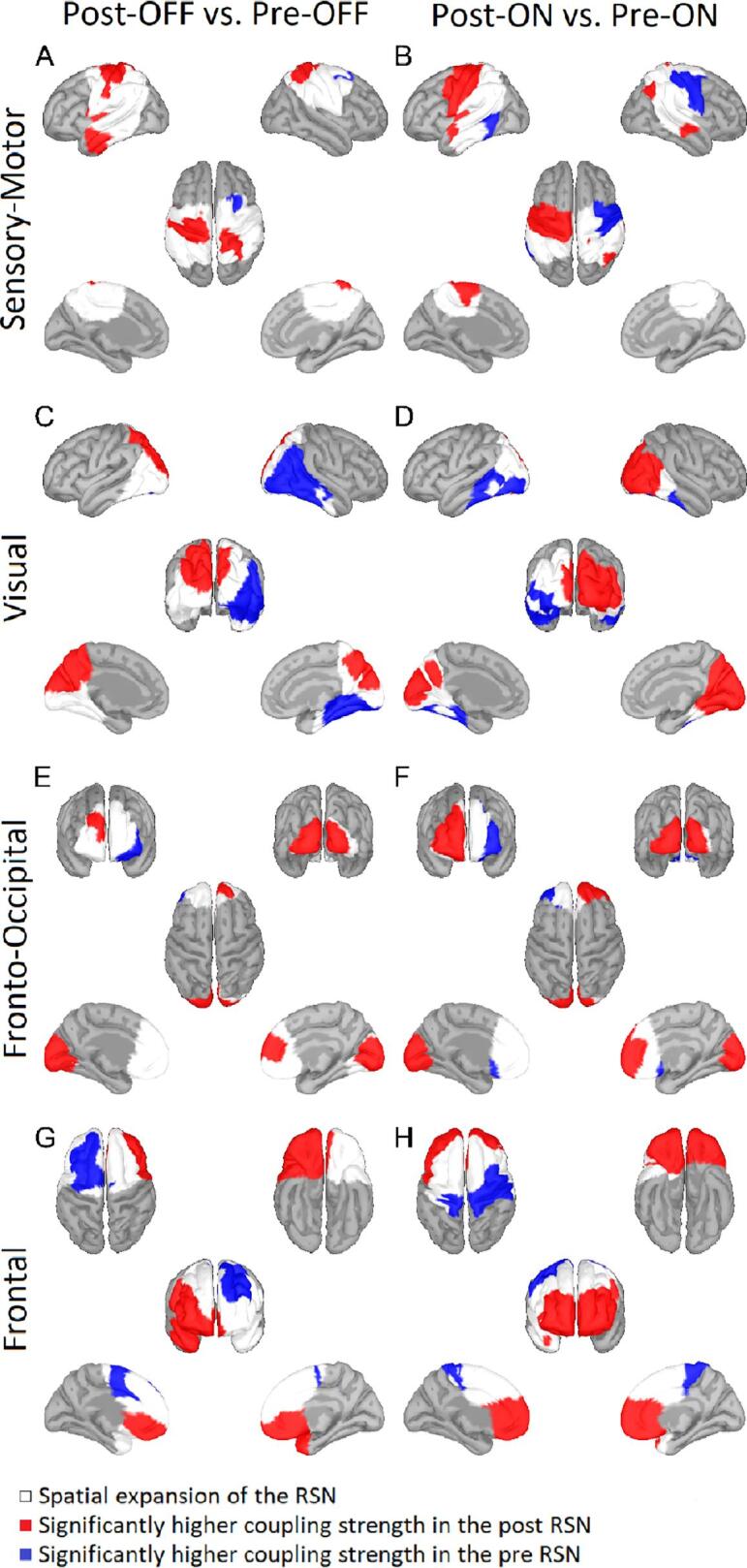
Comparison of resting-state networks before and after electrode implantation The comparison of resting-state networks (RSN) post-OFF vs pre-OFF (left column) and post-ON vs pre-ON (right column) revealed significant differences for the sensory-motor (first row), visual (second row), fronto-occipital (third row), and frontal (bottom row) RSN. Areas belonging to either RSN in post- and/or pre-recording are marked in white. Only areas of the RSNs where the coupling strength in one of the two conditions was at least 0.4 are displayed. Red indicates a significantly higher coupling strength in post-recordings, while blue denotes a significantly higher coupling strength in pre-recording. Significance is given at a p-value below 0.05 after Bonferroni correction for the number of vertices, networks, and conditions. For the sensory-motor RSN, the comparisons post-OFF vs pre-OFF (A) and post-ON vs pre-ON (B) revealed significantly stronger coupling in post-recordings at the somatomotor area. Significant differences between the post-OFF vs pre-OFF conditions (C) and the post-ON vs pre-ON (D) conditions for the visual RSN were present at the visual cortex and the temporal lobe. For the fronto-occipital RSN, significant differences between the post-OFF vs pre-OFF conditions (E) and post-ON vs pre-ON conditions (F) were found, especially in the frontal region of the network at the frontal pole and in the occipital region at the occipital gyrus. For the frontal RSN, the comparison of the post-OFF vs pre-OFF conditions (G) and the post-ON vs pre-ON conditions (H) revealed significantly higher coupling strength at the frontal gyrus during post-recordings. A higher coupling strength in pre-recordings is present OFF medication at the left frontal gyrus and ON medication at the bilateral pre and postcentral gyrus. (For interpretation of the references to color in this figure legend, the reader is referred to the web version of this article.)

For the sensory-motor RSN, both in the OFF- and ON-medication condition the post-recording showed higher coupling strength in the pre and postcentral gyri mostly near the longitudinal fissure but also in the anterior parts of the temporal lobe (Fig. 4 A and B). This alteration in the coupling strength was bilateral when patients were OFF medication and primarily left-lateralized during ON medication conditions. In contrast, the pre-recording showed a higher coupling strength in the right supplementary motor area, which extended to the pre and postcentral gyri when patients were ON medication.

**Fig. 5:**
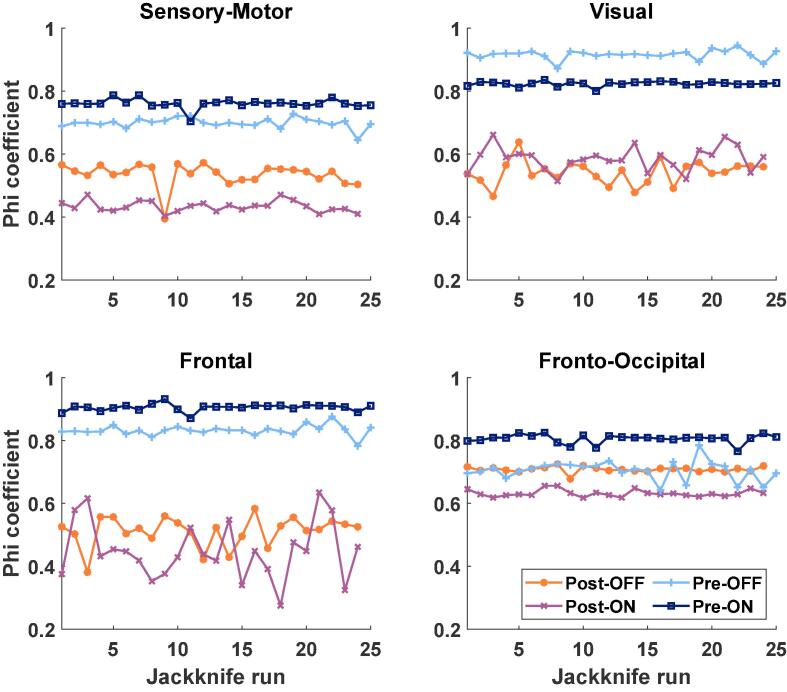
Spatial correspondence between HC-RSNs and PD-RSNs The spatial correspondence calculated with the phi-coefficient based on the thresholded and then binarized coupling strength between the resting-state networks (RSN) of PD conditions (post-OFF, line indicator: asterisk; post-ON, x; pre-OFF, +; pre-ON, square) and the healthy controls is plotted for each jackknife run (x-axis). In case of the pre-surgery results there were 25 jackknife runs and in case of the post-surgery results there were 26 runs.

Regarding the correspondence between PD and HC sensory-motor RSN, the spatial overlap was higher prior to surgery than after (Fig. 5; ON-Medication: pre Phi = 0.761 +- 0.015 [mean +- std], post Phi = 0.433 +- 0.018; p = 8.0e-26; t = 69.43; df = 23; OFF: pre Phi = 0.699 +- 0.016, post Phi = 0.535 +- 0.036; p = 3.55e-14; t = 21.17; df = 23).

### 3.4 Visual network

For the visual RSN, there was increased coupling mainly in the bilateral cuneus and the left parietal lobule for the post-recording compared to the pre-recording (Fig. 4 C and D) and decreased coupling at the right lateral occipital lobule and right temporal lobe during OFF-medication. During ON-medication, an increase in coupling strength for the post-recording was present at the bilateral cuneus and the right occipital and parietal lobule, whereas the coupling strength at the left occipital lobule and left temporal lobe was decreased.

Finally, the spatial overlap between the PD visual RSN and HC RSN was also higher for the pre- than the post-condition (Fig. 5; ON-Medication: pre Phi = 0.824 +- 0.007, post Phi = 0.585 +- 0.039; p = 1.0e-17; t = 30.56; df = 23; OFF: pre Phi = 0.916 +- 0.015, post Phi = 0.542 +- 0.038; p = 1.2e-21; t = 45.64; df = 23).

### 3.5 Fronto-occipital network

In both the frontal and the occipital parts of the FON, there were differences between pre- and post-recordings (Fig. 4 E and F). Specifically, coupling strength was increased in the recording post surgery- compared to the pre-surgery in the right frontal gyrus and bilateral lateral occipital lobule for both medication states. In addition, increases in coupling strength in the right orbitofrontal gyrus were observed during the ON medication state. For the pre-recording, increased coupling strength was present regardless of medication status in the left frontal and orbitofrontal gyrus.

For the FON, we only found a significantly higher overlap with the HC RSN for the pre-condition compared to the post-condition ON Medication (Fig. 5; ON-medication: pre Phi = 0.806 +- 0.014, post Phi = 0.632 +- 0.011; p = 1.37e-21; t = 45.31; df = 23; OFF: pre Phi = 0.705 +- 0.031, post Phi = 0.708 +- 0.009; t = 0.41; df = 23).

### 3.6 Frontal network

For the frontal RSN, we found significant differences between the post- and the pre-recordings in both medication states (Fig. 4 G and H). During the OFF medication state, a significantly higher coupling strength for the pre-recording session was present in the left superior frontal gyrus. In contrast, coupling strengths were higher in an area encompassing the bilateral anterior cingulate gyrus, left frontal and orbitofrontal gyri up to the right temporal pole during post-recording sessions. In regard to the ON medication state, we identified a higher coupling strength for the post- compared to the pre-recording bilaterally in the orbitofrontal and frontal gyrus. In contrast, coupling strength increased in the pre-recording within the pre and postcentral gyri.

Finally, the PD frontal RSN had a higher spatial overlap with the HC RSN before electrode implantation both OFF and ON medication (Fig. 5; ON-medication: pre Phi = 0.905 +- 0.011, post Phi = 0.449 +- 0.092, p = 3.23e-15; t = 23.61; df = 23; OFF: pre Phi = 0.832 +- 0.017, post Phi = 0.511 +- 0.048; p = 4.2e-18; t = 31.78; df = 23).

### 3.7 Comparison of low and high PAC frequencies

Using a 3-factor ANOVA (factors: RSN, medication state, and electrode implantation), we examined whether the low- and high-frequencies of the maximal PAC values changed. We found significant main effects for both low- and high-frequencies. For the low-frequencies, we identified a significant main effect for the factor RSN (F(3,392) = 7.50; p = 6.8e-5; η^2^p = 0.054), as well as for the factor electrode implantation (F(1,392) = 4.58; p = 0.033; η^2^p = 0.012). No significant main effect was present for the factor medication state. Post-hoc tests revealed that the low-frequency in the pre-ON recording was higher for the sensory-motor RSN compared to the visual and the FON RSN (p < 0.05). For the high-frequencies, a significant main effect for the factor RSN (F(3,392) = 11.58; p = 2.7e-7; η^2^p = 0.081) was present in the pre-ON condition, with significant post-hoc tests indicating higher frequencies for the sensory-motor RSN compared to the other three RSNs (p < 0.05).

When analyzing the spectral power before and after electrode implantation, it differed significantly for some cortical regions within the five frequency bands considered. Delta and theta band power was higher after electrode implantation (sup. Fig. 1; |t| > 4.07, df = 145) in the OFF medication condition. For the ON medication condition delta band power was higher after electrode implantation and in alpha band before implantation (sup. Fig. 2; |t| > 4.09, df = 146). In addition, power increased in the beta band in the pericalcarine cortex after electrode implantation, and power decreased in the caudal middle frontal gyrus. Cortical areas with significant power changes were spatially located partly within and outside the considered RSN regions (sup Fig. 3). Regarding the peak frequencies, for the gamma band the peak frequency shifted to a lower peak frequency for only two cortical regions (right pericalcarine cortex and right isthmus of the cingulate gyrus) after electrode implantation OFF medication and ON medication the peak frequency was downshifted for the beta band only in the left posterior cingulate gyrus (OFF: sup. Fig. 4; |t| > 4.09, df = 145 and ON: sup. Fig. 5; |t| = 4.17, df = 146). Again, without a clear spatial relation to the RSNs.

## 4 Discussion

In the present paper, we demonstrate that cortical RSNs from PD patients are altered by the mere insertion of a DBS electrode into the STN before any electrical stimulation is applied. At the same time, RSNs in the pre-operative state more closely resembled the ones of healthy controls.

### 4.1 DBS surgery alters RSNs due to the stun effect

Interestingly, we only found a significant reduction in motor symptoms assessed by the UPDRS-III after DBS surgery in the OFF-medication state but not in the ON-medication state, which could be due to medication already significantly reducing the UPDRS-III, rendering further improvement minor and more difficult to detect. Therefore, the changes in RSNs OFF-medication between pre- and post-recordings could reflect the PD severity measured by the UPDRS-III score (Skorvanek et al., 2017). Nevertheless, we detected alterations in the RSNs between pre- and post-recordings also ON-medication, suggesting that the stun effect affects brain communication subthreshold too, or is not necessarily related to clinical improvement. This finding is in line with the results of Mueller et al., 2020, Mueller et al., 2018, who found different brain connectivity patterns between PD patients treated with DBS or dopaminergic medication. The association with clinical outcomes may also be very specific. For example, using simultaneous MEG-DBS recordings in PD patients, Boon et al. observed that rigidity and bradykinetic symptoms correlated with the whole-brain functional connectivity patterns, including connections between cortical and subcortical sensorimotor regions, albeit these patterns did not relate to tremor symptoms (Boon et al., 2020). However, as we could detect significant changes in all four RSNs of interest, this suggests a wide-spread influence of the stun effect and supports the report of the stun effect not being limited to motor function (Rozanski et al., 2012). Furthermore, the alteration of RSNs in disparate functional domains could be a predictor of side effects induced by STN-DBS (Tamma et al., 2002).

### 4.2 Alterations in different functional RSNs

Within the sensory-motor RSN the coupling strength increased after electrode implantation, especially in the somatomotor area. This finding is compatible with increased excitability of the supplementary motor area (Casarotto et al., 2019) and increased motor cortex activity after dopamine administration (Burciu and Vaillancourt, 2018). Similarly, motor effects induced by DBS correlated positively with functional connectivity, especially in the sensorimotor cortices (Boon et al., 2020). This increased coupling is mirrored by clinical improvement after DBS surgery as measured with the UPDRS-III and potentially due to the stun effect. As the stun effect is associated with an improvement of motor symptoms in PD patients, it was expected that the stun effect would also be reflected in changes of the sensory-motor RSN. As the UPDRS-III from pre- to post-recordings was significantly reduced OFF- but not ON-medication, we surprisingly found similar alterations OFF- and ON-medication between post- and pre-RSNs. This could indicate that medication had a stronger effect than the stun effect on the UPDRS-III, i.e., masking in the clinical assessment of the stun effect, while the more subtle brain wide network effects were still discernible even with medication.

Assuming that the stun effect and DBS are improving PD symptoms, focusing on the visual RSN, the increased coupling within V1 could be associated with the reported shortening of saccade latency under DBS (Temel et al., 2008) and that a shorter saccade latency also leads to a faster response of V1 neurons (Kim and Lee, 2017). Interestingly, however, the sole electrode insertion can increase saccade latency (Antoniades et al., 2012), which fits the reduced coupling strength we found in the visual RSN. These opposing results indicate that the stun effect can lead to the obvious alterations known in DBS, such as the improvements in UPDRS-III, but can also lead to other less obvious alterations, which may be early indications of possible side effects of DBS. Nevertheless, it is striking that the better overlap of the visual RSN for the pre- than post-recording with the HC network was only evident ON– and not OFF-medication because dopaminergic medication in PD patients did not affect cognitive vision tasks (line-, object-, facial-discrimination, visual working memory, and object rotation; Anderson and Stegemöller, 2020). It is possible that this is a task-specific finding that is not transferable to resting activity.

In the frontal and occipital parts of the FON, we also observed alterations after electrode implantation. As this network is based on direct anatomical connections via the fronto-occipital fasciculus (Forkel et al., 2014), it is likely that this fiber tract is functionally affected by the stun effect (Pantazatos et al., 2012). Such a disturbance of the fronto-occipital fasciculus could be due to the spatial proximity to the trajectory of the DBS electrode. However, the cortical areas of the network are clearly separated from the trajectory, as is the visual RSN. Yet they are altered by the stun effect. Only the sensory-motor and frontal RSN encompassed anatomical areas near the trajectory of the DBS electrode. This underscores that the influence of the stun effect on cortical RSNs is not limited to the vicinity of the DBS electrode. In addition, it is well understood that the cortex is connected to the STN, i.e., the target of the DBS electrode, via the basal ganglia-cortical (BGC) loop (Deffains and Bergman, 2019, McGregor and Nelson, 2019). Already the implantation of the electrode in the STN leads to a change in metabolism in parts of the BGC loop (Pourfar et al., 2009), which may influence cortico-subthalamic communication. Together, with the notion that the BGC loop can be functionally subdivided (McGregor and Nelson, 2019), these data might provide potential explanations for the various network changes associated with electrode implantation.

The frontal RSN in our study corresponds to the default mode network detected in previous electrophysiology studies (Brookes et al., 2011, Mantini et al., 2007) and has been associated with self-referential mental activities (D’Argembeau et al., 2005). Thus, the alterations of the frontal RSN due to the implantation of the electrodes align well with the stun effect influencing cognitive functions (Le Goff et al., 2015). In addition, the higher coupling for the pre-recordings could indicate a possible disruption of cognitive networks due to electrode implantation.

### 4.3 Higher correspondence of pre-surgery RSNs with healthy RSNs

Surprisingly, even though the UPDRS-III score was lower during the post-recording compared to the pre-recording, the pre-RSNs had a higher spatial correspondence based on a threshold-constrained coupling strength with the HC-RSNs compared to the post-RSNs. However, in a previous study, RSNs ON-medication – a state with a low UPDRS-III score – had a better correspondence with the RSNs of healthy controls than RSNs OFF-medication, which corresponds to a higher UPDRS-III score (Schneider et al., 2020). Consistent with this finding, the overlap between the PD pre-recording and HC-recording was higher ON-medication than OFF-medication for the sensory-motor, frontal, and FON RSN (p < 1.0e-8). Based on the improved UPDRS-III score we expected even higher overlaps of the RSNs in the post-recording compared to the HC-RSNs than those observed in the pre-recording. This opposing trajectory suggests that the stun effect triggered by inflammatory processes and edema (Chen et al., 2006, Mann et al., 2009), not only modulates the RSNs as shown here, but also masks the potential link between RSNs and UPDRS-III motor symptom severity.

This conjecture is supported by the UPDRS-III score being unrelated to any of the investigated factors. Most surprisingly there was no link between the UPDRS-III and sensory-motor RSNs, albeit motor symptoms improved after electrode implantation. Future studies should determine whether RSNs alterations remain after the stun effect has subsided, or whether alterations of the RSNs under the stun effect have predictive power for the effect of DBS, which may ultimately prove useful for determining an initial stimulation settings for DBS.

### 4.4 Low frequency components are altered by electrode implantation

The low and high frequencies corresponding to the maximal PAC value at each vertex differed between networks. In particular, if the mean low frequency of the maximal PAC value of a RSN was higher in network A than in network B, this was concomitant with the high frequency of the maximal PAC value. This allowed the RSNs to be discriminated based on the low as well as the high frequencies of the maximal PAC values. However, electrode implantation only changed the low frequencies of the maximal PAC value, but not the high frequencies. Nevertheless, because comparable RSNs were found before and after electrode implantation, this suggests that the networks are robust to moderate shifts in frequencies. Furthermore, even though the shifts in the low-frequency band did not result in the RSNs vanishing, spatial changes in coupling strength were evident simultaneously. Since the high frequencies were not changed, this could imply a more important role of the low frequencies in network formation. Similar results were found for a motor task (Jech et al., 2012) and in a resting-state fMRI study that found amplitude changes of low-frequency fluctuations in many brain regions (Luo et al., 2021).

No significant main effect was found for the factor electrode implantation at the higher frequencies, nor was there a significant difference in the power of high frequencies before and after electrode implantation. However, low and high frequencies were modulated when DBS was turned on (Cao et al., 2017). This could indicate that the stun effect is weaker than the DBS effect, or alternatively, that the stun effect attenuates with increasing frequency, which is consistent with the more wide-spread propagation of low frequencies. This aligns well with our findings that electrode implantation cortically modulates mainly the low frequencies of the maximal PAC value.

Especially in the delta band power increased after electrode implantation and ON medication, only three cortical regions showed a decrease in beta band power. Overall, this suggests a frequency-specific modulation of power by electrode implantation. In addition, this could influence the observed differences in RSNs, although we assume a minor role for two reasons. First, power changes were not spatially specific with respect to the RSNs. If the power changes were to shift the low-frequency component of the PAC signal significantly, this would have been expected to occur in the spatial region of the RSNs. Second, the power changes in the delta/theta band occurred mainly OFF medication, although changes due to electrode implantation were detected in RSNs in both medication conditions. Furthermore, the shifts of the peak frequencies occurred so sparsely that a substantial influence on the power or the frequency components of the PAC signal is not to be expected.

### 4.5 Limitations

A caveat of our study is that it is impossible to eliminate all potential confounders. Since the second measurement usually takes place the day after surgery, patients may still be fatigued, generally weakened, or also affected by anesthesia. This is relevant because recovery of cognitive abilities after anesthesia takes time (Mashour et al., 2021), and fatigue can have an influence on cortical networks (Zhang et al., 2020). To prevent fatigue, patients could take breaks between measurement blocks. Since increased alpha power has been associated with increased fatigue (Craig et al., 2012), we calculated the alpha power in both lateral occipital sulci, the inferior, and superior parietal lobule based on the Mindboggle atlas. We did not find a significant change in alpha power between the first and last run for any of the four conditions or selected brain areas (|t| < 2.22, df: post recordings = 25; pre recordings = 23) and between conditions (|t| < 3.18, df: post OFF vs ON = 150; pre OFF vs ON = 142; OFF post vs pre = 146; ON post vs pre = 146).

The long measurement duration combined with increasing fatigue also poses the risk of the head moving away from the sensors over time. However, before the start of each measurement block, the position of the head was adjusted if necessary. Then the position of the HPI coils was determined at the beginning of each measurement run, which did not allow a continuous position verification. However, the relative position of the HPI coils to the MEG sensors along the z-axis did not significantly differ between the individual measurement runs of a measurement condition (|t| < 2.51, df: post recordings = 25; pre recordings = 23) and between conditions (|t| < 2.30, df: post OFF vs ON = 150; pre OFF vs ON = 142; OFF post vs pre = 146; ON post vs pre = 146). Thus, it can be assumed that the individual measurement blocks for each condition provided comparable data quality.

Besides the aforementioned limitations of conducting MEG measurements closely following electrode implantation (e.g., < 3 days), earlier measurements do offer a clear advantage. Since the impact of the stun effect decreases over days to weeks following DBS implantation (Maltête et al., 2008, Singh et al., 2012), the impact of the stun effect on the present data is presumed to be the most severe. At a later time point, the stun effect might not be detectable, not even in the UPDRS-III score. As a result, longitudinal measurements designed to track the stun effect decay and its subsequent impact on RSNs would be necessary to better describe the consequences of the stun effect.

## 5 Conclusion

The stun effect of DBS electrode implantation induces changes within different functional RSNs of PD patients that are not spatially limited to the vicinity of the DBS electrode trajectory. These effects are partially modulated by the state of dopaminergic medication. In line with our main hypothesis, the stun effect did not only influence the sensory-motor network, but also other functional networks. However, contrary to our hypothesis, stun effect-induced changes in RSNs exhibited no clear-cut relationship to induced clinical improvements in the UPDRS-III. Although stun effect-induced RSN changes partially matched RSN changes due to dopamine or DBS reported in other studies, the missing link to the UPDRS-III indicates that the stun effect is not restricted to clinically assessed PD symptoms. Our hypothesis for this missing link is that inflammatory processes caused by electrode implantation alter brain activity extensively but not strongly enough to induce changes in specific functional networks and general clinical scores, i.e., the UPDRS. Further insights might be obtained when investigating these RSNs after the stun effect has subsided and under the influence of DBS. Such an analysis could reveal the connection between stun effect and DBS effects, in addition to whether the RSNs measured directly after DBS surgery can be used to choose a first DBS setting and estimate side effects.

## Declaration of Competing Interest

The authors declare that they have no known competing financial interests or personal relationships that could have appeared to influence the work reported in this paper.

## Data Availability

Data will be made available on request.
