# Supplementary material for: Alterations of resting-state networks of Parkinson‘s disease patients after subthalamic DBS surgery
Source: Neuroimage Clin. 2023 Jan 4;37:103317. doi: 10.1016/j.nicl.2023.103317 (PMC9850202; doi:10.1016/j.nicl.2023.103317)
Supplement: Supplementary data 1 [file mmc1.docx]

**Supplementary Material:**

Figure 1: Altered spectral band power OFF medication by electrode implantation

For 62 different cortical regions, based on the Mindboggle atlas, significant differences in power are displayed for five different frequency bands before and after electrode implantation in the dopaminergic medication OFF condition. Blue indicates a significant decrease in power after electrode implantation, and red indicates a significant increase. An independent t-test followed by Bonferroni correction for the 62 cortical regions, 5 frequency bands, and 2 medication states was used.

Figure 2: Altered spectral band power ON medication by electrode implantation

For 62 different cortical regions, based on the Mindboggle atlas, significant differences in power are displayed for five different frequency bands before and after electrode implantation in the dopaminergic medication ON condition. Blue indicates a significant decrease in power after electrode implantation. No significant increase was found. An independent t-test followed by Bonferroni correction for the 62 cortical regions, 5 frequency bands, and 2 medication states was used.

Figure 3: Cortical regions with altered power after electrode implantation.

Cortical regions with significant differences in power in at least one of five frequency bands (delta: 1-4 Hz, theta: 4-8 Hz, alpha: 8-12 Hz, beta: 12-35 Hz, gamma: 35-100 Hz) emerge in the comparison of post-OFF vs. pre-OFF (three left columns) and post-ON vs. pre-ON (three right columns). Brain areas with significant changes are highlighted. The four resting-state networks (RSN; rows 1 & 2: sensory-motor, rows 3 & 4: visual, rows 5 & 6: fronto-occipital, rows 7 & 8: frontal) are shown for spatial comparison. Areas belonging to either RSN in post- and/or pre-recording are marked in white. Only areas of the RSNs where the coupling strength in one of the two conditions was at least 0.4 are displayed.

Figure 4: Altered peak frequencies OFF medication by electrode implantation

For 62 different cortical regions, based on the Mindboggle atlas, indicates whether the peak frequency of five different frequency bands differs significantly before and after electrode implantation in the dopaminergic medication OFF condition. Blue indicates a significant decrease in power after electrode implantation. No significant increase was found. An independent t-test followed by Bonferroni correction for the 62 cortical regions, 5 frequency bands, and 2 medication states was used.

Figure 5: Altered peak frequencies ON medication by electrode implantation

For 62 different cortical regions, based on the Mindboggle atlas, indicates whether the peak frequency of five different frequency bands differs significantly before and after electrode implantation in the dopaminergic medication ON condition. Blue indicates a significant decrease in power after electrode implantation. No significant increase was found. An independent t-test followed by Bonferroni correction for the 62 cortical regions, 5 frequency bands, and 2 medication states was used.
