# Supplementary figures and images for: Alterations of resting-state networks of Parkinson‘s disease patients after subthalamic DBS surgery
Source: Neuroimage Clin. 2023 Jan 4;37:103317. doi: 10.1016/j.nicl.2023.103317 (PMC9850202; doi:10.1016/j.nicl.2023.103317)

Spectral Band Power: Pre-ON vs. Post-ON

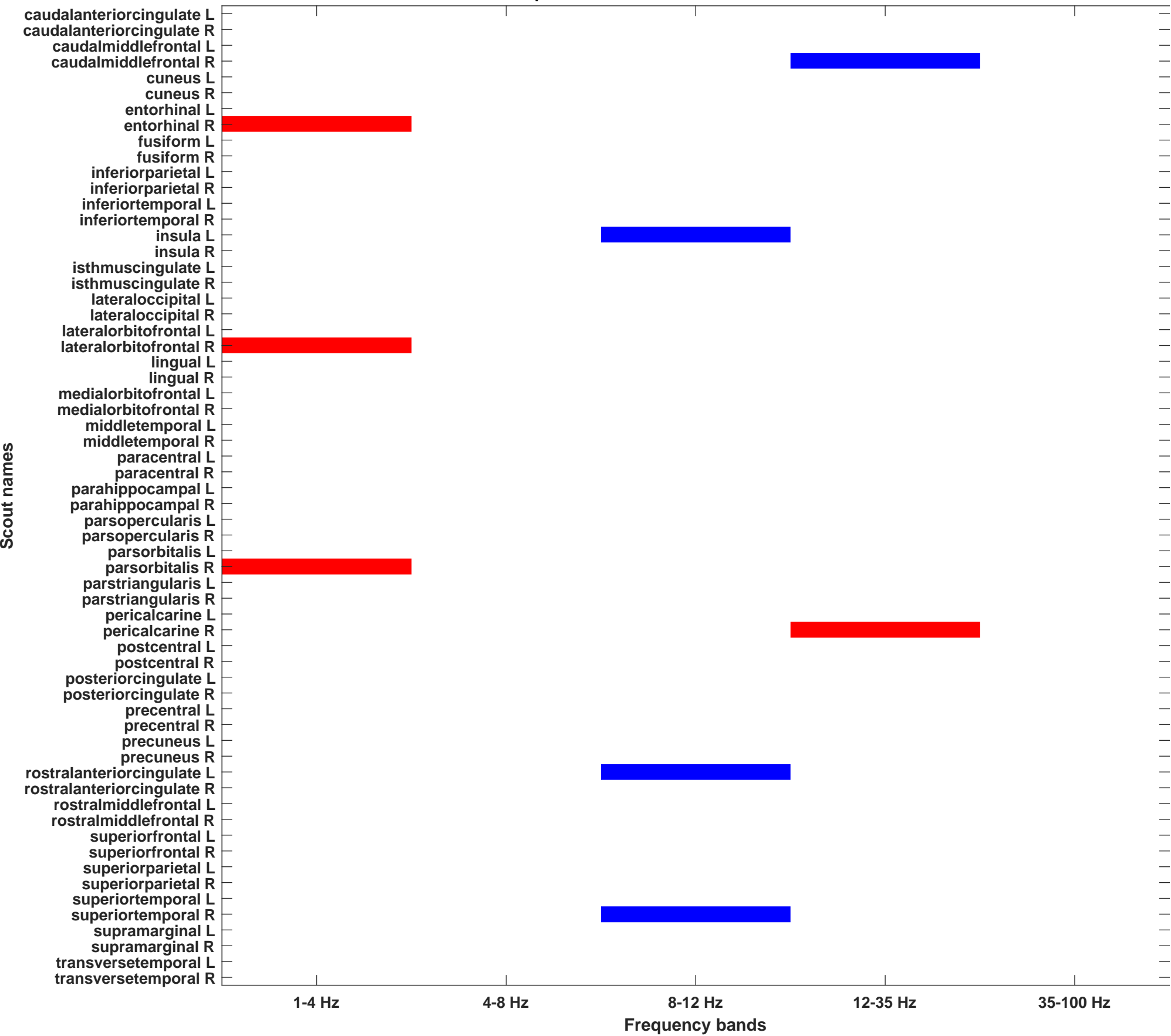

Supplement: Supplementary data 3 [file mmc3.pdf]

Post-OFF vs. Pre-OFF

Post-ON vs. Pre-ON

Sensory-Motor

Visual

Fronto-Occipital

Frontal

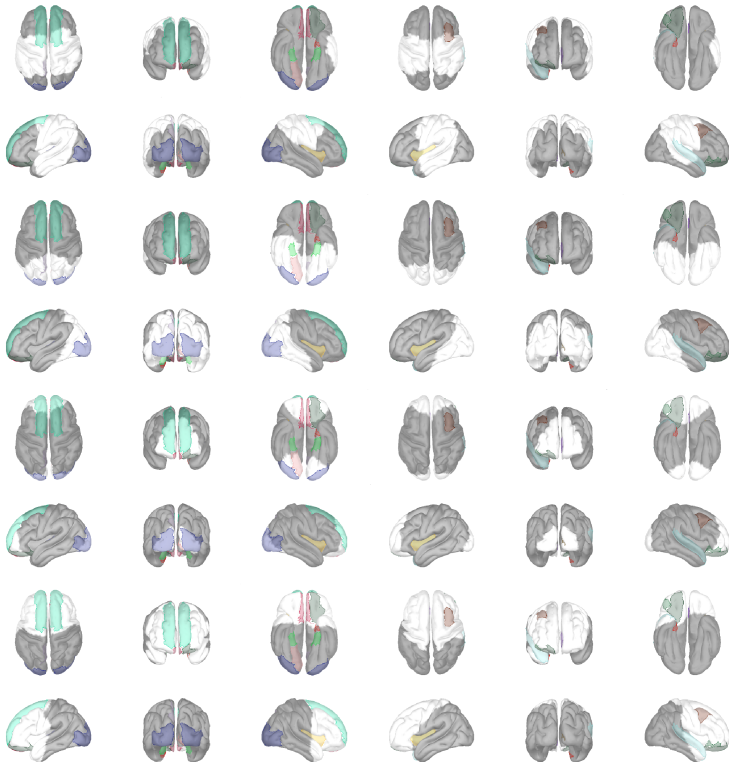

Supplement: Supplementary data 4 [file mmc4.pdf]
