# Supplementary material for: Alterations of resting-state networks of Parkinson‘s disease patients after subthalamic DBS surgery
Source: Neuroimage Clin. 2023 Jan 4;37:103317. doi: 10.1016/j.nicl.2023.103317 (PMC9850202; doi:10.1016/j.nicl.2023.103317)
Supplement: Supplementary data 5 [file mmc5.pdf]

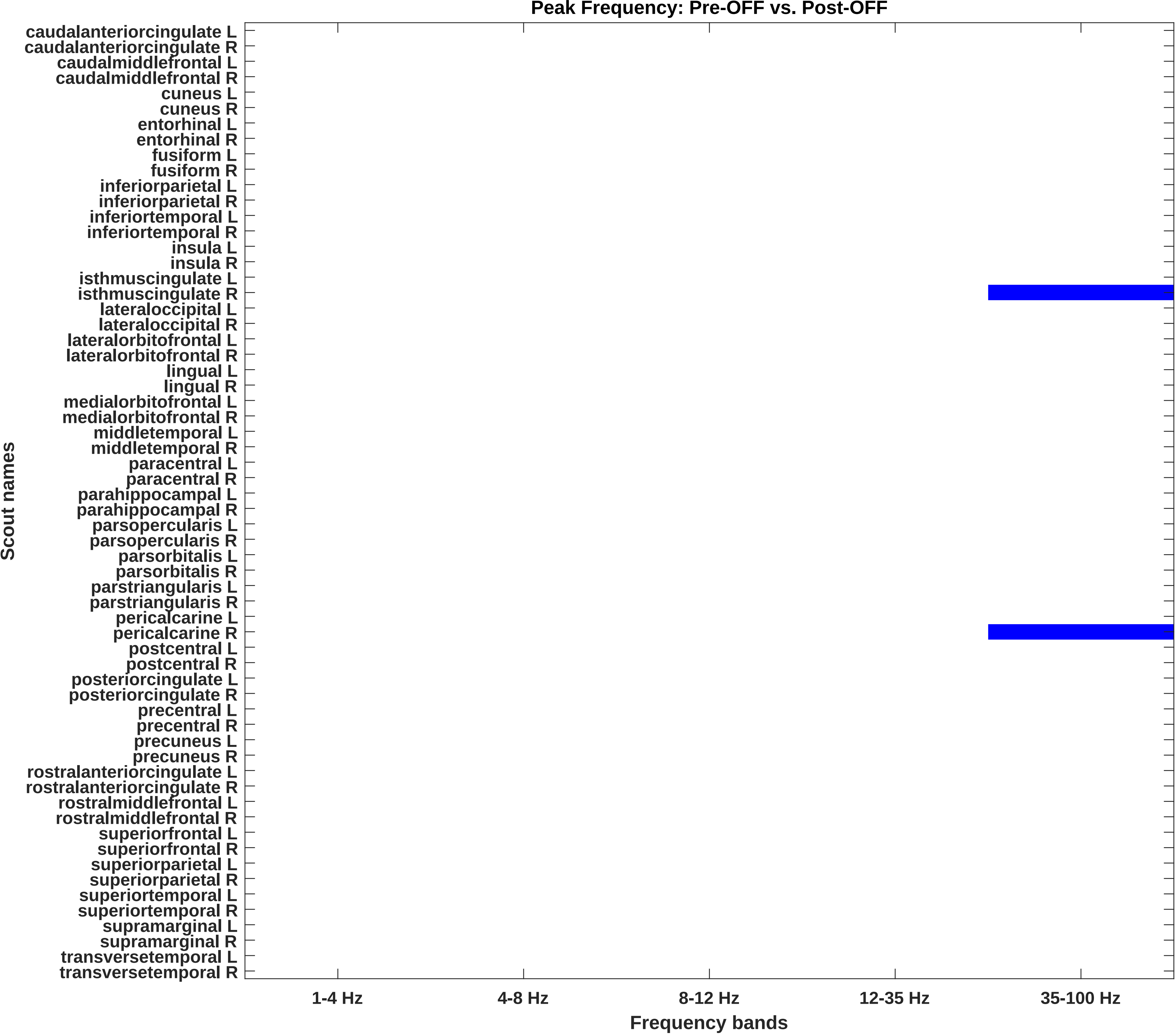

Peak Frequency: Pre-OFF vs. Post-OFF
caudalanteriorcingulate L
caudalanteriorcingulate R
caudalmiddlefrontal L
caudalmiddlefrontal R
cuneus L
cuneus R
entorhinal L
entorhinal R
fusiform L
fusiform R
inferiorparietal L
inferiorparietal R
inferiortemporal L
inferiortemporal R
insula L
insula R
isthmuscingulate L
isthmuscingulate R
lateraloccipital L
lateraloccipital R
lateralorbitofrontal L
lateralorbitofrontal R
lingual L
lingual R
medialorbitofrontal L
medialorbitofrontal R
middletemporal L
middletemporal R
paracentral L
paracentral R
parahippocampal L
parahippocampal R
parsopercularis L
parsopercularis R
parsorbitalis L
parsorbitalis R
parstriangularis L
parstriangularis R
pericalcarine L
pericalcarine R
postcentral L
postcentral R
posteriorcingulate L
posteriorcingulate R
precentral L
precentral R
precuneus L
precuneus R
rostralanteriorcingulate L
rostralanteriorcingulate R
rostralmiddlefrontal L
rostralmiddlefrontal R
superiorfrontal L
superiorfrontal R
superiorparietal L
superiorparietal R
superiortemporal L
superiortemporal R
supramarginal L
supramarginal R
transversetemporal L
transversetemporal R
Scout names
1-4 Hz
4-8 Hz
8-12 Hz
12-35 Hz
35-100 Hz
Frequency bands
